# Solomon Charles Smith

**Published:** 1903-04-11

**Authors:** 


### The Hospital.

A Journal of

The Medical Sciences and Hospital Administration.

Edited by Sir Henry Burdett, K.C.B.

Vol. XXXIV.—No. 863. [April 11, 1903.

### In Memoriam

### SOLOMON CHARLES SMITH, M.D., M.R.C.P.,

Late Co-Editor of "The Hospital."

It is with feelings of the deepest regret that we announce to our readers the death of Dr. Solomon Smith, co-editor of The Hospital. Dr. Smith had been in bad health for some months, and a few days ago it was found necessary to operate; but his strength, reduced by a long and trying illness, was not sufficient to enable him to rally from the shock, and he passed away peacefully at Walton-on-Thames on Sunday last, April 5th. Dr. Smith had contributed to the columns of The Hospital since 1894, and in 1896 he permanently joined the staff as co-editor with Sir Henry Burdett, K.C.B. Since that date he devoted his entire energies to promoting the interests of The Hospital. His wide experience, great general knowledge, calm and sound judgment, and devotion to work rendered him an ideal occupant of the editorial chair of The Hospital. He kept closely in touch with all modern developments of medicine, and whilst the judicial trend of his mind caused him to be in sympathy with a high standard of medical etiquette, his wide general knowledge and experience enabled him to consider with an open mind any innovations in practice or procedure which occurred in the medical world at home or abroad. He was a man of marked integrity; and although on most subjects he held definite views, he never allowed these to interfere with his judgment of individual instances occurring in the course of his work. He was an able and convincing writer. By his fellow-workers on the editorial staff of The Hospital he was regarded with both affection and respect. He was ever ready to help and advise, and no matter was too small to claim his attention or disturb his patience. His loss is personally regarded as irreparable by his co-editor and those of the editorial staff who were closely associated with him. Dr. Solomon Smith received his medical education at Birmingham, Guy's Hospital, and in Paris. He became M.R.C.S.Eng. in 1863, L.S.A. 1865, L.R.C.P.Edin. 1872, M.D.Durham 1882, and M.R.C.P.Lond. 1892. He practised for some years in Halifax, where he held the appointment of Surgeon and, finally, Consulting Surgeon to the Halifax Royal Infirmary. He retired from this post in 1892 and came to London. Finding that a life without definite employment was unsuited to one of his active mind, he became physician to the Westminster General Dispensary, and occupied himself with medical journalism, first on the editorial staff of the "British Medical Journal" and later on that of The Hospital. He resigned his post at the Westminster General Dispensary in 1902. He was a fellow of the Royal Medico-Chirurgical Society and of the Medical Society of London; member of the Pathological, Clinical, and Harveian Societies, and of the British Medical Association; and formerly President of the Leeds Medico-Chirurgical Society. Dr. Solomon Smith made many useful contributions to medical literature on various subjects, being the author of "Epidemiology of Cholera" in "Allbutt's System of Medicine," "Waterborne Diseases," "XXth Century Practice of Medicine," and numerous articles in the medical periodicals.

Dr. Solomon Smith will be buried at Halifax, the place of his birth, and the scene of a long and useful medical and social career.

